# Interventions to improve the person-centered quality of family planning services: a narrative review

**DOI:** 10.1186/s12978-018-0592-6

**Published:** 2018-08-28

**Authors:** Nadia Diamond-Smith, Ruby Warnock, May Sudhinaraset

**Affiliations:** 1 Department of Epidemiology and Biostatistics, Global Health Sciences, University of California, San Francisco, 550 16th Street, 3rd Floor, San Francisco, CA 94158 USA; 2 University of California, Los Angeles, USA

## Abstract

Person-centered care, a key component of quality of care, is receiving increased attention for maternal and reproductive health. While many interventions have aimed to improve person-centered care for family planning, there is no known narrative review of person-centered-focused interventions in family planning and the outcomes of these interventions. This narrative review fills this gap by conducting a rigorous analysis of interventions that address person-centered care and measure family planning related outcomes, including quality, knowledge and use/continuation. The search of the published and grey literature, from 1990 to 2015 identified 5530 papers, of which 25 were ultimately included in the analysis (after exclusion criteria was applied). We grouped these interventions under seven domains of person-centered care: dignity, autonomy, privacy/confidentiality, communication, social support, supportive care, and trust. We find that person-centered interventions had high success in improving perceptions of quality and knowledge of family planning among clients; however, results were less consistent in improving family planning uptake and continuation. These findings will help program and policy makers develop interventions that incorporate person-centered components to have the highest likelihood for success in improving clients’ experiences and family planning use.

## Plain English summary

Person-centered care is a component of quality of care that moves beyond clinical quality of care to include concepts such as support, respect, and autonomy. While there has recently been increased attention to person-centered aspects of care for delivery services, there has been little attention paid to this component of quality for family planning. Additionally, little is known about what types of interventions have aimed to improve this component of quality, and whether they have been at improving client’s experiences and family planning related outcomes. The aim of this narrative review is to, first, explore what types of interventions have been conducted in the past that focused on improving person-centered quality. The second aim is to understand what impact these interventions have had on client’s experiences, and outcomes such as family planning knowledge, uptake and continuation. We conducted a systematic review of papers published between 1990 and 2015, and identified an initial 5530 papers, of which 25 were ultimately included in the analysis, which we then analyzed in a narrative fashion. Based on an existing framework, we grouped these interventions by the focus of their approached, into seven domains: dignity, autonomy, privacy/confidentiality, communication, social support, supportive care, and trust. Overall, the interventions that measured perceptions of quality (such as satisfaction) and people’s knowledge of family planning led to improvements in these outcomes in most cases. However, interventions that measured family planning uptake and continuation had mixed results in improving those outcomes. This narrative review adds to our understanding of both the types of interventions aiming to improve person-centered quality of family planning care, and suggests that while there is fairly good evidence that family planning knowledge and client’s experiences are positively impacted by such approaches, more research is needed to understand the impact of person centered care interventions on family planning uptake and continuation.

## Background

Person-centered care is critical for ensuring quality family planning services. Person-centered care has gained increased attention in the past decade, including in the developing world [1, 2]. Person-centered care refers to the range of interactions that places the person at the center of all clinical decisions –including their strengths, future plans and rights [1]. It encompasses a move to see beyond a patient to understand the person as a whole. It extends beyond patient’s experiences of care to include specific care processes that may improve women’s experiences of care and better reflects women’s preferences and values related to family planning. Past studies suggest that across the world, women experience poor patient-provider interactions, are ignored, berated and yelled at, and discriminated against during care [3–5]. Some literature also suggests that person-centered aspects of quality such as information-sharing and interpersonal relations are correlated with increased adoption and continuation of modern family planning methods [6]. A recent study found that a higher quality of interpersonal care, as measured by women’s reports and observation of providers, was associated with increased contraceptive use [7]. Postpartum family planning and counseling, offered face-to-face within a facility following delivery and childbirth, has been associated with increased adoption and maintenance of contraception up to seven months [8]. It is important to understand if interventions aiming to improve person-centered aspects of family planning care are associated with person-centered and other health outcomes, such as contraceptive use and uptake.

In the context of women’s health, much of the literature on person-centered care has primarily been conceptualized in the field of maternal health, including delivery care and childbirth [9–12]. For reproductive health services broadly, Sudhinaraset et al. define domains for person-centered health care through the Person-Centered Care Framework for Reproductive Health [13] including dignity (i.e. receive care in respectful and caring setting), autonomy (i.e. involving women in decision-making), privacy/confidentiality, communication with providers/patients, social support in the facility including family members, supportive care (i.e. timely, compassionate and caring manner of care), trust in providers, and health facility environment. In the framework, there is a bidirectional relationship between provision of care and person-centered care. For this paper, we define person-centered care for family planning by this same framework: care that enhances and ensures dignity, autonomy, privacy/confidentiality, communication, social support, supportive care, and trust. We did not include the domain related to a safe environment because other work has looked at this domain and our focus was on the interpersonal aspects of quality.

Preliminary strategies to improve person-centered maternity care outlined by the World Health Organization (WHO) include social support through a companion of choice, mobility, access to food and fluids, confidentiality and informed choice, assuring high quality information for women, and high quality of provider standards [14]. Much less is known about strategies to address poor person-centered care for family planning, or other reproductive health needs. Part of this may be due to a lack of awareness about the importance of person-centered care, and part to lack of consensus on the definition. As is clear from the discussion above, person-centered care is multi-faceted and encompasses a broad range of domains. Interventions that address person-centered aspects of family planning quality may not use that terminology, thus making understanding the evidence on the impact strategies to improve person-centered quality for family planning on various outcomes challenging. To complicate things further, interventions that address a person-centered component of quality of family planning care do not always measure a person-centered outcome (such as experiences or satisfaction) and rather sometimes measure family planning uptake or continuation (for example). Thus we are left to hypothesize about whether or not the experience is on the pathway between person-centered interventions and other outcomes of interest. In this manuscript, we distinguish between person-centered care *processes* (i.e. dignity, autonomy, privacy/confidentiality, communication with providers/patients, social support in the facility including family members, supportive care, and trust in providers) and person-centered *outcomes* (i.e. patient satisfaction and experiences). Given these complexities, we take a broad definition of person-centered interventions for family planning, where interventions had to focus on at least one aspect of person-centered process as we have defined it. We included interventions that did and did not measure person-centered quality as an outcome; those that did not measure a person-centered quality outcome had to have a family planning related outcome.

While there exist reviews of interventions aimed to improve the quality of family planning broadly, there are no known reviews on interventions to improve person-centered care for family planning. This has created a knowledge gap, and risks scholars replicating the same interventions and not being able to build off the successes (and learn from the challenges) of previous work. This review seeks to fill this gap by providing a comprehensive review of past strategies and interventions conducted that aimed to improve person-centered care for family planning services. This will help policy makers and practitioners design and implement programs and interventions that build on past experiences and apply tested strategies. Specifically, our objectives are to: 1) describe interventions related to person-centered care, including context and populations; and 2) identify effects that person-centered care interventions have had on family planning uptake, continuation, and person-centered quality of care, from the client’s perspective. More broadly, our focus is on understanding and describing what types of person-centered interventions for family planning have been conducted, and exploring whether, in general, these have led to changes in person-centered or family planning related outcomes. The goal is not to rigorously assess the quality of interventions or research, nor to provide estimates of impact, given the wide scope of the topic and subsequent heterogeneity in intervention approaches and outcomes.

## Methods

We followed conduct and reporting standards for systematic reviews of social interventions set forth by the Campbell Collaboration [15], including the development and publication of a protocol with pre-determined inclusion criteria and analysis plan which was registered with the PROSPERO International prospective register of systematic reviews [16].

### Inclusion and exclusion criteria

Inclusion criteria for the review included: 1) an evaluation of a person-centered intervention; 2) facility-based intervention; 3) family planning outcome as either clinical or person-centered; 4) scientific rigor of study design; 5) abstract in English; 6) studies published between 1990- September 2015.

We defined “person-centered” as interventions that addressed the domains outlined by Sudhinaraset et al.: dignity, autonomy, privacy/confidentiality, communication, social support, supportive care, and trust. See *Appendix 1. Database Search Strategy* for a specific list of search terms.

We defined “facility-based” as having some linkage to facility based family planning care (not existing solely in the community – for example, mass media campaigns). Interventions needed to have a person-centered component of family planning care.

Outcomes could be either clinical (i.e. increased family planning uptake, decreased unintended pregnancies) or person-centered (i.e. client experiences, satisfaction). Outcome measures had to focus exclusively on family planning clients and not on providers, in order to be also person-centered. In terms of our analytical framework, we hypothesized that person-centered care interventions would lead to improvements in clinical or person-centered care outcomes. Therefore, in order to improve outcomes, interventions first needed to address a person-centered care component (i.e. system and provider responsiveness, patient engagement with a health facility, patient-provider communication, interpersonal treatment, and the range of advice, outreach, follow-up, respect and dignity, and emotional, instrumental, or informational support).

Studies needed to be rigorous in nature. We defined this to include experimental and quasi-experimental studies that included a facility-based, person-centered, family planning intervention. Quasi-experimental quantitative studies needed to collect longitudinal and/or cross-sectional data from treatment and comparison groups. We excluded studies without a valid control or comparison condition.

This review includes only quantitative evidence. Although we planned to do an integrated mixed-methods review including both quantitative and qualitative evidence, there were no qualitative studies in our search results that met our criteria in terms of person-centered interventions with useable data. Our inclusion criteria for qualitative evidence was that studies must report individual narratives from participants and must include discussion of factors that determine individual’s participation in, and benefits from, person-centered quality programs.

Included studies were limited to those with an abstract in English published between 1990 and September 2015. We chose a start date of 1990 because (1) the Bruce-Jain Framework in 1994 led to more focus on quality in family planning and thus we thought most evaluations would be since that time and (2) the nature of family planning programs has changed in the past decades and going farther back seemed less relevant. Study participants had to be women of reproductive age (15–49 years), male adopters of family planning at any age and/or providers of family planning services.

### Search strategy

We systematically searched the published literature in PubMed, CINAHL, and EconLit using controlled search terms and free-text terms combining three main components: (a) family planning services (b) person-centered care and (c) intervention terms. The search strategy can be found in *Appendix 1. Database Search Strategy*. We supplemented our database strategy with an extensive range of searches in electronic databases, grey literature, relevant journals, and organization websites. Grey literature sources searched included dissertations, theses, government reports, nongovernmental organization reports, and funder reports using search engines and databases. We also performed keyword hand searches and contacted key personnel of relevant organizations for recommended studies. We used bibliographic back referencing of captured reviews and included studies to identify additional studies that met our search criteria. All searches were conducted during September 2015. A search diary was maintained describing the search methods, keywords used, and a tally of search results.

### Screening, data extraction and critical appraisal

One member (RW) of the study team assessed titles and abstracts for inclusion, after a preliminary process of double coding (two researchers coding the same abstracts) and reaching consensus about our approach for inclusion. Two researchers then independently performed the full-text review and extracted information from each quantitative study included in the review using a pre-determined data extraction form. The standardized form had the following domains: study setting, sample characteristics, objectives, design, data collection and analysis methods, and conclusions. In addition, outcome measures and results were extracted from all studies.

**Fig. 1:**
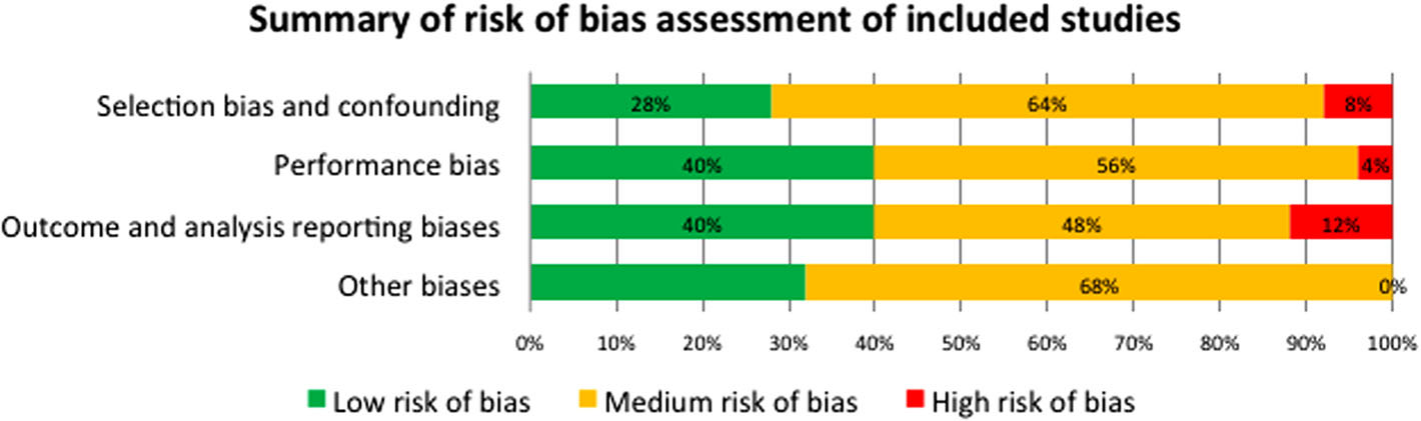
Summary of risk of bias of included studies

Two researchers (RW and NDS) then independently assessed the risk of bias of all included quantitative studies using an adaptation of an established and verified set of criteria, to assess risk of bias in experimental and quasi-experimental studies [17, 18]. The critical appraisal tool contained 71 criteria to assess the risk of these biases: (1) selection and confounding, (3) performance, (2) outcome and analysis reporting, and (4) other biases. We coded the studies as low, medium, or high risk of bias for each of the four types of bias based on the risk of bias assessment. All disagreements about inclusion, data extraction, and critical appraisal were resolved through discussion and involvement of a third independent member of the team when necessary (Fig. 1). As can be seen in Fig. 1, the majority of studies in our review were deemed to be of low or medium risk of bias.

### Analysis

Since meta-analysis of the quantitative studies was not possible due to heterogeneity in their measured outcomes, we present a narrative synthesis using descriptions of study characteristics, outcome measures, and key findings. We summarize overarching themes and consistency of directions of outcomes for interventions and measures that share common characteristics. Similar approaches have been recommended and used elsewhere [10, 19, 20]. Discussions between team members were used to build consensus on the narrative synthesis.

Outcomes were classified into three categories: (1) family planning related = family planning uptake, continuation, intention to use, pregnancy, etc.; (2) person-centered care related = satisfaction, measures of quality of care, patient/provider interactions, changes in self-efficacy or power, etc.; and (3) knowledge related: knowledge of methods, fertility, etc.

## Results

### General overview

The database searches yielded 3660 articles, and the hand searches yielded an additional 2273 articles. After duplicates were removed, title-abstract screening was performed for 5530 articles. Full texts were reviewed for 372 potentially eligible studies. These studies came from database searches (*n* = 280), hand-searches of websites (*n* = 51), bibliography back referencing (*n* = 30), and theses and dissertations search (*n* = 11).

Of the 372 studies identified in the full-text review, we excluded 314 studies after applying the exclusion criteria. The following were the main reasons for exclusion: the study did not meet our criteria of an evaluation of a person-centered intervention (*n* = 90); the study was not a facility-based person-centered quality intervention (*n* = 199); the study was not examining quality of delivery, family planning and abortion services with family planning person-centered or clinical outcomes (*n* = 21); the study was not of appropriate rigor (*n* = 4). Among the remaining 58 full-text articles, an additional 33 studies were excluded, for the following reasons: the study was not a facility-based person-centered quality intervention (*n* = 8); the study was not examining quality of delivery, family planning and abortion services with family planning person-centered or clinical outcomes (*n* = 6); the study was not of appropriate rigor (*n* = 19).

**Fig. 2:**
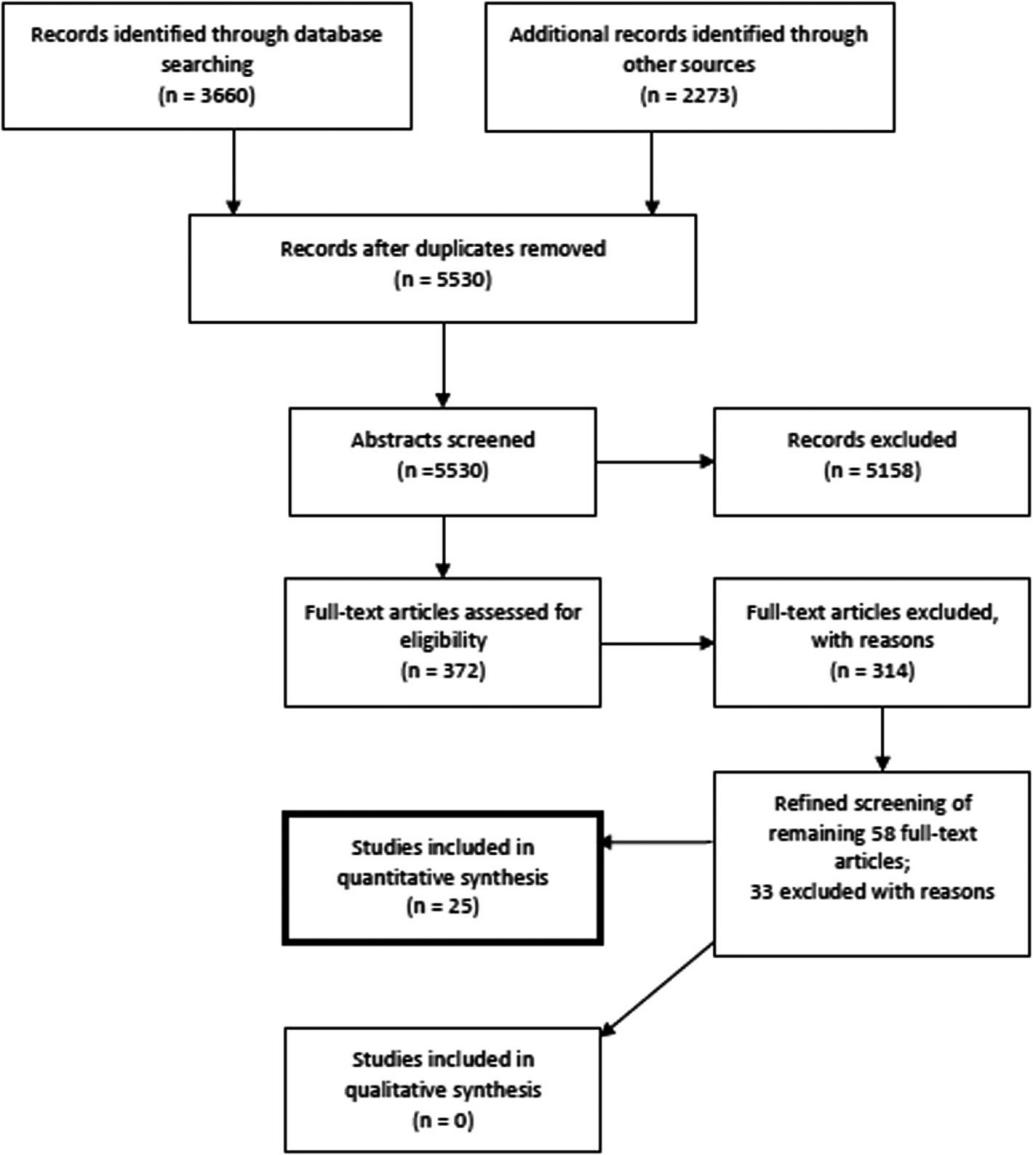
Study search flow diagram

After the second round of exclusions, 25 studies were included (Fig. 2).

### Description of included studies

**Fig. 3:**
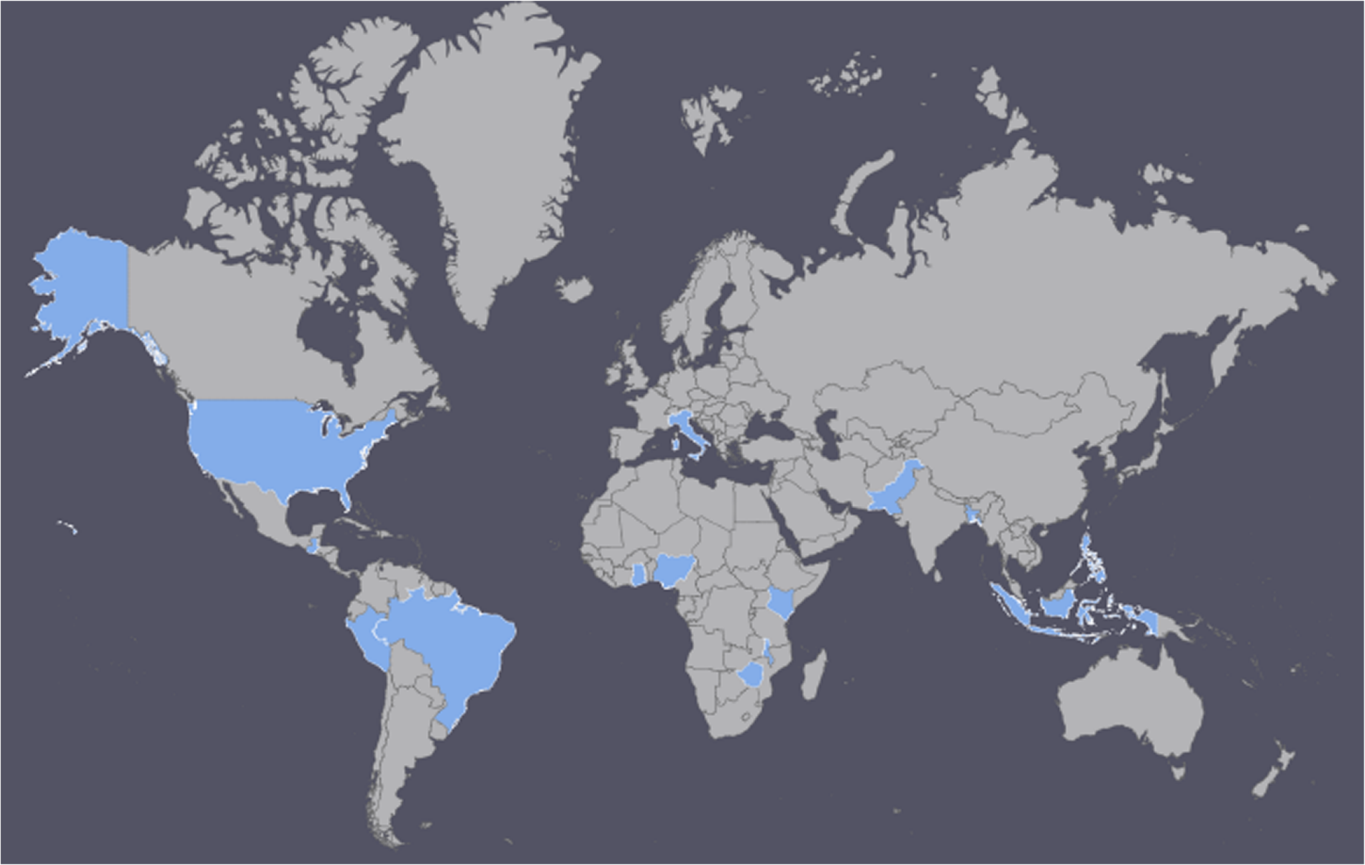
Map of included studies

A total of 25 studies were included in the final analysis. Two of the papers were analyzing different outcomes in the same intervention (Costello et al. [21], Jain et al. [22]). For the remainder of this paper, they will be considered one study and all outcomes included. Eleven of the studies were conducted in the United States or Europe, five were conducted in Africa, five in the Asia/pacific region, and 3 in South or Central America (Table 1, Fig. 3).

**Table 1:** Summary of included studies (F*p* = Family planning related; PCC = person-centered care related; Knowledge = knowledge related outcomes)

Study, year	Target Population, Country, sample size	Description of intervention	Study design	PCC Domains	Findings
Amatya et al. 1994 [23]	Husbands, Bangladesh *N* = 617	Provided counseling to the husbands of women who received NORPLANT about the method.	Quasi-experimental design [prospective pilot study]	Communication, Social Support	FP: Lower discontinuation rates at 36 months (hazard of discontinuation 0.81 higher in control), PCC: no difference in satisfaction
Bensussen-Walls & Saewyc 2001 [42]	Adolescents (pregnant, aged 13–18), United States, *N* = 106	Comprehensive, interdisciplinary teen-centered prenatal care clinics (Young Women’s Clinic & Teen Pregnancy and Parenting Clinic) were developed to help out-of home, high risk, parenting and pregnant teens. These clinics had public health nurses, social workers, dieticians, midwives, and adolescent focused providers. The providers received training in providing care to adolescents.	Retrospective chart review and a case-comparison design	Dignity, Privacy/ confidentiality, Communication	FP: More family planning use at 8 weeks (87.7% of teen clinic clients were using a contraceptive method compared with 64.3% of adult clinic clients)
Berenson & Rahman 2012 [31]	Low income young women (16–24 women, sexually active, not pregnant, making < 30,000/year), United States, *N* = 1155	The intervention consisted of one- on- one counseling for 45 min. Counselors used educational and behavioral techniques based on the health belief model. Clients were also given handouts with simple instructions. The counselor reviewed the instruction verbally and helped the patient develop a cue to remember the pill, as well as discussion other birth control and pregnancy related information. An additional arm tested this intervention plus a weekly follow-up phone call until the clients started the method and then monthly for 6 months. In the calls, counselors gave instructions, discussed side effects, and clients had a 24-h emergency number they could call.	Randomized controlled trial	Privacy/ confidentiality, Communication	FP: No impact on OC at 3, 6 or 12 months, condom, pregnancy rates. Comparing Intervention vs. Standard at 12 months: OC: 20% vs. 20%; condoms at last intercourse: 31% vs. 29%.
Carneiro Gomes Ferreira et al. 2011 [32]	Post abortion (1–2 weeks post abortion), Brazil, *N* = 246	Face-to-face contraceptive counseling lasting 30 min was provided. This session covered individualized counseling, provided guided information based on past experiences, myths and beliefs about contraception, and free provision of family planning and verification of their knowledge about how to use it.	Randomized controlled trial	Privacy/ confidentiality, Communication	FP: Increase in FP uptake; probability of continuation at 6 months greater in the intervention group (41% higher in the intervention group)
Charron-Prochownik et al. [28] 2013	Adolescent girls (13–19) with type 1 or 2 diabetes, United States, *N* = 109	The intervention consisted of additional video/book based information over 3 visits. The fist was a DVD, which provided evidenced based information, the second also a DVD with exercises and the third a book reinforcing information from the first DVD.	Randomized controlled trial	Privacy /confidentiality, Communication	Knowledge: Increased knowledge about family planning (FP) (group by time interactions F[6, 81.5] = 10.41, P, 0.0001), FP: More intention to use FP (F[6, 534] = 3.40, *p* = 0.0027) and talk to provider about FP (F[6, 82.4] = 2.56, *p* = 0.0254)
Costello et al. 2001 [21]	Women (new-users, received method in last 6 months), Philippines, *N* = 869 intervention and *N* = 859 control	Providers and their supervisors were trained to help clients meet their self-defined reproductive needs. Providers were trained in information exchange (relevant and accurate information and providing high quality services) and supervisors were trained in providing support to providers.	Quasi-experimental design	Autonomy, Communication	PCC: Reported better quality of care (large number of quality indicators)
Danielson et al. 1990 [34]	Adolescent boys (15–18 who had ambulatory care at participating hospital), United States, *N* = 971	A 30-min slide tape presentation with explicit photographs and information about reproductive anatomy, fertility, HIV/STIs, contraception, and other topics was developed and provided to clients. After watching the slide-tape, clients received a consultation guided by the client’s interests. A patient centered approach guided the counseling (non-judgmental, modeling and rehearsing conversations with partners, etc.).	Quasi-experimental design	Dignity, Privacy/ confidentiality, Communication	FP: Reduced sexual activity among those who never had sex (OR = 1.31, *p* < 0.001); increase method use (OR = 1.51, *p* < 0.05) Knowledge: increase in knowledge of fertility/family planning (various indicators)
Exner et al. 2009 [24]	Men (referred from their female partners), Nigeria, *N* = 149 Intervention, *N* = 132 comparison	Seven models to promote dual-protection were delivered in two 5-h workshops, 1 week apart. Topics included HIV stigma and knowledge, pregnancy risk, risk reduction strategies, facilitating sexual negotiation, challenging gender based violence, and setting and implementing risk reduction goals. Communication, assertiveness, and negotiation skills were emphasized, and different methods such as small group discussions, songs, proverbs, role-playing and games, were utilized.	Quasi-experimental design	Dignity, Autonomy, Communication	PCC: Great safer sex efficacy (0.17, *p* < 0.05), less relationship response to condom use (− 0.19, *p* < 0.05) and less interpersonal power (− 0.16, *p* < 0.05). FP: Greater intention for future consistent condom use (OR = 2.11, *p* < 0.05), greater intention to use condoms consistently (0.23, *p* < 0.01). Lower odds of unprotected sex (OR = 0.34, *p* < 0.01), greater odds of condom use at last sex (OR = 4.10, *p* < 0.001), lower odds of refusal to use condom with main partner (OR = 0.28, *p* < 0.01).
Gilliam et al. 2014 [27]	Women (ages15–29), United States, *N* = 60	A theory-based app was developed using human centered design. The app was based on the theory of planned behavior, addressed gaps in LARC knowledge and provided information on other methods, was designed for a variety of learning styles, had unbiased and evidence based content and could be used in the clinic setting.	Randomized controlled trial	Dignity, Privacy/ confidentiality, Communication	Knowledge: Significantly higher knowledge of contraceptive effectiveness (2 out of 3 measures) FP: Increased interest in the implant (6.5 to 29.0%, P,< 0.02). PCC: Users were highly satisfied (no comparison)
Jain et al. 2012 [22]	Women (new users), Philippines, *N* = 1728	Providers and their supervisors were trained to help clients meet their self-defined reproductive needs. Providers were trained in information exchange (relevant and accurate information and providing high quality services) and supervisors were trained in providing support to providers.	Quasi-experimental design	Dignity, Communication, Supportive Care	FP: No significant effect on modern family planning use or unintended birth; PCC: Impact of a number of quality indicators (needs assessed, method choice, information received, interpersonal relations, continuity of care)
Kim et al. 2000 [33]	Women, Indonesia *N* = 233	The Smart Patient intervention occurred while patients were waiting for their appointment in the waiting room. Patients were led through three exercises on a leaflet that encouraged patients to ask questions. The second part of the intervention had patients think through what they wanted to ask the nurse (using a list of common questions as a prompt) and then write them down. In the final step, the patient could practice asking her questions.	Quasi-experimental design	Autonomy, Communication, Supportive Care	PCC: Clients’ ratings of self-expression (4.0 to 4.2, *p* < .0001) and satisfaction increased (4.2 to 4.4, *p* < 0.0001); no effect on clients’ perspectives on the counseling experience
Kim et al. 2003 [41]	Women recruited from 64 clinics in two districts, only new users, Indonesia, *N* = 768	A 5-day workshop for providers emphasized client-centered counseling and skills including rapport setting, encouraging dialogue and decision-making. Additional arms included (1) providers doing a self-assessment and (2) self-assessment plus peer review meetings (every week for 16 weeks).	Quasi-experimental design	Autonomy, Privacy/ confidentiality	FP: The discontinuation rate at 8 months was lower, but the difference was only marginally significant (life table, X^2^ = 2.99, *p* = 0.08).
Kraft et al. 2007 [25]	Couples (women ages 18–25 and their primary male partner), United States, *N* = 223	Partners Against Risk-Taking: A Networking, Evaluation and Research Study (PARTNERS) included a 3 session intervention with women and their male partners, in groups of up to 6 couples. The intervention addressed psychosocial and relationship factors related to preventive strategies such as family planning and HIV/STIs.	Randomized controlled trial	Dignity, Autonomy, Communication, Social Support	FP: No effect on contraceptive uptake; PCC: improvement in the psychosocial variable measuring positive expectations pertaining to partner’s support for contraception (F = 0.483, *p* = .029) and participation in decision-making about FP (*F* 27.15, *p* .001)
Langston et al. 2010 [44]	Post abortion (women 18 years or older who do not want to become pregnant right away), United States, *N* = 380	The intervention assessed a WHO developed tool called the Decision-Making Tool for Family Planning Clients and Providers. It includes a double-sided flip chart with information for providers on one side and clients on the other side. Providers were also trained to encourage patients to ask questions and write down questions for their provider.	Randomized controlled trial	Dignity, Autonomy, Supportive Care	FP: No impact on choosing a very effective method, initiation, or use at 3 months.
León et al. 2004 [35]	Women (new adopters), Peru, *N* = 215	Providers received a 2-day training on the job aids assisted Balanced counseling strategy, with an additional 1-day re-training.	Quasi-experimental design	Dignity, Communication, Supportive Care	Knowledge: Knowledge of IUD/hormonal methods chosen higher (*p* < .05, one-tailed) FP: continuation and switching rates did not differ, reproductive goals more likely to be met (*p* < .01).
León et al. 2005 [36]	Women, Guatemala, *N* = 320	Balanced counseling uses 2 techniques to simply the client’s experience of choosing a family planning method. The first is to do a needs assessment to help the provider focus on methods that are appropriate for the client given her needs or situation. The provider then only describes these methods. The second technique involves the use of visual aids that help both the provider and client.	Quasi-experimental design	Dignity, Communication, Supportive Care	PCC: Improved Quality of Care (1 tailed t-value: 13.81, *p* < 0.001), increased session length (3.94, *p* < 0.001)
Nobili et al. 2007 [38]	Post abortion, Italy, *N* = 186	Counseling was provided by psychologist or gynecologist and lasted for 30 min. The intervention consisted of a semi-structured interview to understand the women’s needs, the offer of information and education about methods, and then choosing a method and checking for understanding.	Randomized controlled trial	Dignity, Communication	Knowledge: Knowledge (Z = − 3.91, *p* = .0001), favorable attitudes towards contraception (Z = − 3.81, *p* = .0001) FP: use of effective contraception increased (65% to 80%, *p* = .0002, no change in control group)
Petersen et al. 2007 [39]	Women (ages 16–44, at risk of unintended pregnancy), United States, *N* = 764	Participants received pregnancy and STI prevention counseling, followed by a booster session 2 months later. The counseling session was based in motivational interviewing and emphasized three elements: discrepancies between pregnancy intention and contraceptive use, sharing information, and promoting behaviors to reduce risk. Counseling was tailored based on baseline data collected on clients and focused on increasing self efficacy and effective use. Women could also obtain or get a referral for a method.	Randomized controlled trial	Dignity, Autonomy, Communication	FP: No significant differences
Rawlins et al. 2013 [45]	Women (receiving reproductive health services: ANC, PNC, FP, and L&D), Malawi, *N* = 139	A performance and quality improvement intervention was conducted over a three-year period to improve family planning, as well as delivery, antenatal and post natal care.	Quasi-experimental design	Supportive Care	PCC: Higher scores on client assessments (difference in means, *p* < 0.001), but not for counseling
Reynolds et al. 2008 [43]	Women (FP, MCH, or STI/HIV clients), Kenya, *N* = 30	Based on the findings of a quality improvement cycle, a training package for supervisors was developed. The developed package consisted of a one week training with supervisors on improving performance, leading teams, skills required of being a supervisor, etc.	Quasi-experimental design	Supportive Care	PCC: No improvements in client satisfaction
Sarnquist et al. 2014 [26]	Women (18–40 years old, HIV- positive, seeking ANC), Zimbabwe, *N* = 33 standard-of-care (SOC) and *N* = 65 intervention participants	The intervention consisted of three 90-min group sessions (or about 12 women each) aimed to increase FP use and negotiating power. The sessions used a variety of learning techniques such as discussion, behavior modeling, songs, and role-play.	Quasi-experimental design	Dignity, Autonomy, Communication	PCC: Increased control over condom use (t-test, *p* = .002), increased relationship power (*p* = .01), Knowledge: increased knowledge about IUDs (*p* = .002), FP: No change in intent to use a condom or use of a method increased relationship power
Sathar et al. 2005 [40]	Women seeking Family planning in 1 district, Pakistan *N* = 381 baseline, *N* = 443 end line	Trained providers to focus on meeting client needs through a more patient-centered approach, and that included addressing the client’s gender and power situation at the household level. Used a framework to guide the providers (salutation, assessment, help, and reassurance).	Quasi-experimental design	Dignity, Supportive Care, Trust	PCC: Improved patient provider interaction
Schwandt et al. 2013 [30]	Women (18 years or older, fertile, and wanting to wait at least 12 months before next pregnancy), Ghana, *N* = 684	Group counseling with four main components: “(a) introduction to the basic physiology of reproduction—with an emphasis on the quick return of fertility after an abortion; (b) an overview of family planning and the different methods available; (c) messages tailored to the individual patient to help her determine the correct method for her and the potential side effects with that method and (d) an emphasis on establishing linkages with family planning services in each woman’s locale.”	Quasi-experimental design	Dignity, Autonomy, Communication	Knowledge: No difference in modern contraceptive knowledge
Schwarz et al. 2013 [37]	Acute care women (18–45 years old), United States, *N* = 814	The intervention consisted of a computer kiosk where patients could get information and facilitate access to contraceptives. It provided information and allowed women to request a prescription.	Randomized controlled trial	Privacy/ confidentiality, Communication	FP: More likely to report receiving a contraceptive prescription (16% vs. 1%, *p* = .001); No difference in FP use last sex or knowledge
Winter & Breckenmaker 1991 [29]	Adolescent (younger than 18 years old and high risk for teen pregnancy), United States, *N* = 1256	Services tailored to youth included 1–1 counseling, visual aids, multiple clinic visits, longer appointments, provider training in adolescent development, attention to the comfort of the teen, the encouragement of male participation, support to teens for resisting peer pressure and encouraging parent involvement.	Quasi-experimental design	Dignity, Autonomy, Privacy/ confidentiality, Communication, Social Support, Supportive Care	FP: More likely to use a method at 6 months (*p* < 0.01), more likely to continue method (*p* < 0.05), less likely to become pregnant (*p* < 0.05)

Most (12) of the studies were published between 2000 and 2009, 10 were published after 2010, and 3 between 1990 and 1999 (The two papers from the same study were published in different decades, and thus both are included here).

### Description of interventions and outcomes

We use Sudhinaraset et al.’s domains of person-centered care processes to organize different types of interventions. These include: 1) communication; 2) privacy/confidentiality; 3) supportive care; 4) dignity; 5) autonomy; 6) social support; and 7) trust [13] (Table 2).

1. *Communication*

The majority (19) of interventions focused on increasing information about family planning and reproductive health for patients, usually through some type of counseling or communication. We broke these down into those that increased information broadly (reproductive health/biology, sexually transmitted infections (STIs), as well as family planning) and those that provided personally tailored family planning knowledge/counseling. A few interventions provided broader knowledge about methods and reproductive health to men to husbands of women who just received NORPLANT (Amatya et al. [23]) and to men or women and their partners about HIV and pregnancy/family planning (Exner et al. [24]; Kraft et al. [25]). A few provided information to women about family planning methods that was not tailored specifically to that individual woman’s needs (Sarnquist et al. [26]; Gilliam et al. [27]; Charron-Prochownik et al. [28]; Winter & Breckenmaker [29]). Many studies incorporated both tailored and general family planning/reproductive health information. One used group counseling (Schwandt et al. [30]), others individual counseling face to face (Berenson & Rahman [31], Carneiro Gomes Ferreira et al. [32]; Kim et al. [33]), or multi-media (Danielson et al. [34]). Interventions that focused on personally tailored information, based on an assessment of the client’s needs/situation, included the balanced counseling strategy (León et al. [35] and León et al. [36]), a computer program that the client interacted with to get information that helped providers then tailor the counseling (Schwarz et al. [37]), a focus on patient self-defined needs (Costello et al. [21]; Jain et al. [22]), and an initial semi-structured/motivational interview which then helped the provider focus the counseling sessions (Nobili et al. [38]; Petersen et al. [39]).

Of the 19 interventions that had a communication component, 11 measured family planning uptake or use at last sex. Of these, 5 found higher uptake/use at last sex and 6 found no impact. Six of these interventions measured family planning continuation specifically, and of these, 3 found lower discontinuation and 3 found no difference. Eight of these interventions measure some type of person-centered outcome, and 7 found an improvement in this outcome, and 1 no effect. Seven interventions measured family planning knowledge, 6 finding increased knowledge and 1 no effect. Finally, 2 interventions measured some other related outcome (intention to use, reproductive goals being met) and both had a positive impact.

2- *Privacy/Confidentiality*

We categorized interventions that provided patients information that they could consume on their own (without a provider) as having a privacy component. Some of these gave handouts and leaflets for providers to give patients (Berenson & Rahman [31]; Kim et al. [33]). Some used audiovisual tools such as slide tapes (Danielson et al. [34]) or DVDs (Charron-Prochownik et al. [28]). Other interventions included interactive tools such as a mobile-phone application (Gilliam et al. [27]) and computer assisted intervention (Schwarz et al. [37]). Most of these tools helped guide the provider in providing information to the patient, either actively or more passively (DVDs, handouts). The computer assisted intervention (Schwarz et al. [37]) helped gather information about the patients for the provider before the counseling session so that the provider could provide more tailored information. While a number of interventions provided information in an individual manner or to specific sub-groups (for example, teens), we categorized these under either information or dignity, respectively, although they do also promote privacy.

Of the 6 interventions that addressed this domain, 4 measured family planning uptake/last use and 2 found increases and 2 no impact. Three of the interventions measured continuation and all found no impact. Only 1 in this group measured person-centered outcomes, and it found improvements in these measures. Of the 3 that measured some type of knowledge outcome, all found positive associations, as did the 2 that measured other types of outcomes.

3- *Supportive Care*

Another category of interventions focused on increasing provider skills and thus their ability to provide supportive care. These included giving providers a framework to guide their patient interactions (Sathar et al. [40]) and training them in rapport setting and how to encourage dialoged and shared decision-making (Kim et al. [41]). A related set of interventions focused specifically on addressing provider bias or stigma, thus giving them tools to provide more equitable and non-judgmental care. One such intervention focused on high-risk parenting and pregnant teens in the US (Bensussen-Walls & Saewyc [42]). Another study focused on training providers to understand gender and power dynamics in households in order to provide higher quality family planning care (Sathar et al. [40]). Two interventions trained supervisors (Costello et al. [21]/ Jain et al. [22] and Reynolds et al. [43]). In these interventions, the supervisors gained skills in providing support or better management for their teams. Related to this, other interventions found different ways of reinforcing the training that providers had on counseling or other topics with additional time for re-training or additional training itself (León et al. [35] and León et al. [36]; Reynolds et al. [43]), or having providers conduct self assessments (Kim et al. [41]). Langston et al. [44] gave providers a flip chart with information for providers on one side and clients on the other, and training in helping clients ask questions (Langston et al. [44]). A few interventions included changes in service provision more broadly, such as making clinics more youth friendly across the board (Bensussen-Walls & Saewyc [42]; Winter & Breckenmaker [29]). Two interventions included quality improvement cycles, which happened at the facility level to improve patient-centered quality (Rawlins et al. [45] and Reynolds et al. [43]).

Of the 11 interventions that had components of supportive care, 4 measured family planning uptake, and of these, 2 saw an improvement and 4 no difference. Three measured continuation, with 1 finding a positive impact and 2 no difference. Seven of these intervention measured a person-centered outcome and 6 found a positive impact and one no impact. An additional 1 measured and found a positive impact on knowledge and 1 measured and found a positive impact on another outcome (reproductive goals being met).

4- *Dignity*

We classified interventions as having a dignity focus if they either focused on high risk populations, or on broader societal/cultural factors. Many of the interventions focused on high-risk populations (*N* = 10). A number of interventions targeted adolescents of one or both sexes (Berenson & Rahman [31]; Bensussen-Walls & Saewyc [42]; Charron-Prochownik et al. [28]; Danielson et al. [34]; Kraft et al. [25]; Winter & Breckenmaker [29]). Other interventions focused on high-risk populations of women, including post-abortion clients (Nobili et al. [38]; Langston et al. [44]; Carneiro Gomes Ferreira et al. [32]), or women with HIV (Sarnquist et al. [26]).

Those that aimed to work on social/cultural factors included interventions empowering women to understand gender and power dynamics (Sathar et al. [40]), discussing topics including gender based violence and facilitating sexual negotiation (Exner et al. [24]), focus on increasing negotiating power, and practice doing so using songs, behavior modeling, etc. (Sarnquist et al. [26]), support in resisting peer pressure (Winter & Breckenmaker [29]), and addressing psychosocial and relationship factors (Kraft et al. [25]). Another group of interventions that fell into this category are those that focused on providing information tailored to the specific needs of that client, such as through balanced counseling (León et al. [35] and León et al. [36]) or an individual client centered approach (Schwandt et al. [30] and Petersen et al. [39].

Of the 15 interventions that had a dignity component, 11 measured family planning uptake and 5 found a positive impact and 6 no difference. Four measured continuation and 2 found a positive impact and 2 no impact. Four papers measured and found a positive impact on person-centered outcomes. Five of the studies measured knowledge, with 4 finding a positive impact and 1 no impact. Finally, 3 of these studies measured another outcome, and all found a positive impact (reproductive goals being met, favorable attitudes towards contraception, and intention to use).

5- *Autonomy*

A number of interventions focused on enhancing people’s decision making power and autonomy in choosing a family planning method, and these we classified in the autonomy domain. These included interventions that helped clients write down questions for the provider before the session and even practice asking questions (Kim et al. [33]; Langston et al. [44]), focused on self efficacy and effective use of methods (Petersen et al. [39]), guided clients to meet their self defined needs (Costello et al. [21]/ Jain et al. [22], and supported client decision-making (Kim et al. [41]). In an effort to enable clients to act on their decisions, one intervention helped patients develop cues to remember methods (Berenson & Rahman [31]), while another helped them develop linkages in their community to more easily access methods (Schwandt et al. [30]). Somewhat related to dignity, Sarnquist et al. [26] focused on helping clients build their negotiating power. Finally, Gilliam et al. [27] was based on a theory of planned behaviors, which links an individual’s beliefs to their ability to act.

Of the 10 studies that had a component focused on autonomy, 5 measured family planning uptake, and all five found no impact. An additional 3 measured continuation and all of these also found no impact. Of the 5 that measured person-centered outcomes, all 5 found a positive impact. Three measured knowledge, with 2 finding a positive impact and 1 no impact, and the 1 that measured another outcome found a positive impact (interest in the implant).

6- *Social Support*

A few interventions specifically focused on increasing social support among partners/family members in their family planning decisions and use. One involved engaging partners/husbands alone (Amatya et al. [23]), another worked with couples together (Kraft et al. [25]), and another aimed to encourage youth to involve their parents (Winter & Breckenmaker [29]).

Of the three interventions with social support components, 2 measured family planning uptake, and 1 found positive and 1 no impact. Two also measured continuation, both with positive impact. Finally, 2 measured person-centered care, and 1 found a positive and 1 no impact.

7- *Trust*

Only one intervention specifically mentioned guiding providers in building trust with clients, by giving providers a framework to approach clients with that included guidance on salutation, assessment, help, and reassurance (Sathar et al. [40]), we which defined as specifically addressing components of trust. This intervention only measured person-centered outcomes and found a positive impact on these outcomes.

## Discussion

### Summary of person-centered family planning interventions

This review summarizes the types of interventions that have been used to improve person-centered care in family-planning services, and the effects that these interventions have had on quality of care, patient knowledge, and family planning use. We contribute to existing literature by describing person-centered care interventions and effects on family planning use, continuation, and satisfaction. The domains set forth by Sudhinaraset et al. provide a useful framework for organizing interventions. Of the interventions identified in our search, communication was the most common type of intervention with 19 identified in our review, followed by dignity (*n* = 15), supportive care (*n* = 11), privacy/confidentiality (*n* = 6), autonomy (*n* = 10), social support (*n* = 3), and trust (n = 1). Importantly, all of the studies except for two (which focused on supportive care) represented more than one domain of person-centered care process.

Although concepts of person-centered care are encompassed in the interventions identified here, most do not explicitly state having this approach. Only the study published by Costello et al. [21]/ Jain et al. [22] was based on a stated client-centered approach. Thus, only one of the included studies mentioned being based upon or influenced by a philosophy of person-centered care, hence the person-centered care aspects had to be inferred in the review process from the intervention descriptions.

### Summary of main results

Of the studies identified, only 11 included outcome measures of person-centered care, including patient satisfaction and those related to experiences of care, for example increased control over family planning use and length of time for family planning counselling. Most found improvements in person-centered care outcomes, with only 2 out of the 11 reporting no difference in terms of person-centered care outcomes as result of the intervention [23, 43]. These two interventions were in the communication and supportive care domains (one in both of them). Notably, most studies that measured a family planning knowledge related outcome had a significant improvement. This may be because knowledge is an easier indictor to make an impact on compared to behaviors or farther downstream factors. The only one that did not have a positive impact had a communication and autonomy component.

However, the results for family planning use, uptake or continuation, and changes in fertility-related outcomes were less clear-cut. Within each domain, the papers that measured family planning uptake and continuation were split about 50/50 between no effect and improvements. The primary exception to this was the interventions that had an autonomy component, of which none had a significant effect. The interventions within the privacy domain also all had no impact on continuation. It is possible that changing behavior is more complicated than knowledge or satisfaction, and requires multi-faceted, longer term, or more in-depth types of interventions. As an example of this, Winter & Breckenmaker [29], find that a multi-component intervention that fell into 5 domains (dignity, privacy/ confidentiality, communication, social support, and supportive care) among adolescents resulted in lower rates of adolescent pregnancy. Future interventions should consider addressing more than 1 domain of person-centered care when attempting to impact family planning related outcomes. Additionally, future studies should focus on the long-term impacts of person-centered care processes and health behaviors such as unintended pregnancies, abortions, and fertility rates. Finally, only six of the evaluations did any type of cost effectiveness assessment; while 13 of the studies did not even mention costs. Thus we were unable to include any comparison or discussion of whether person-centered interventions are a cost effective approaches for improving client experiences or family planning outcomes.

### Limitations

The final twenty-five included studies varied greatly in terms of study design and focus, methodology and outcome measures. All interventions had to focus on a component of person-centered family planning care, but, given that we did not restrict on type of outcome measured, there was variability in outcomes, limiting our ability to compare impact. Our definition of person-centered interventions was broad to ensure that we captured as many interventions as possible that addressed this complex idea, however, this does make summarizing the impact of specific types of interventions challenging. Furthermore, most of the studies were in the developed world (US and Europe), and we do not know how appropriate the interventions might be in other settings, or how their impacts might different in a different context. This review provides a descriptive narrative synthesis of the current work in person-centered family planning interventions.

There were challenges amongst the team to concisely define person-centered care and what interventions fit into existing and modified frameworks. Additionally, the framing of interventions is variable across the research, making the task of capturing and summarizing the existing literature especially difficult. Thus, it is possible that some interventions were identified as not incorporating elements of person-centered care because researchers were not using this framework. Future research should work to report interventions with precise language, in order to increase transparency in reporting.

There are several important arguments for including person-centered care into maternal health care; however, there is less literature surrounding person-centered family planning care. There is a need for more person-centered approaches to family planning care. This review process was not restricted to the specific search term ‘person-centered’ and the authors used their judgment and the Sudhinaraset et al. framework to identify the aspects of person-centered care. Included studies did not have to explicitly state that their interventions were based on person-centered care frameworks, and in fact, few identified studies were framed explicitly in this way. As well as testing interventions that include aspects of person-centered care, further research is also needed to study the utility of frameworks of person-centered care.

In addition, we only included studies published in the English language, and this certainly limited our findings. Although other researchers could recreate our search in additional languages, the fact that we are limited to English language results limits the scope of our findings. Finally, while we conducted a risk of bias assessment, we did not do additional study quality assessments or remove studies based on the risk of bias, given our goal to describe the relevant studies identified.

## Conclusion

This narrative review presents rigorously evaluated person-centered family planning interventions. We found that interventions that measured family planning knowledge and person-centered outcomes in general improved these outcomes, however, person centered interventions were less consistent in increasing family planning uptake and use.

This review has several implications for future research. Our description of the quantitative evidence suggests there is a need for more rigorous quantitative studies measuring person-centered outcomes. Future research could use mixed-methodologies to test new rigorous measures of person-centered quality. The lack of qualitative research found by our review highlights a large gap in existing research. Additionally, we ended up excluding many studies that used multicomponent intervention methods due to a lack of rigor in the studies of this type; however, it would have been difficult to include such studies due to unclear correlation of intervention and outcomes in such studies.

Because person-centered family planning interventions are implemented in many different regions globally, it is important for researchers and program implementers to understand that an intervention that works in in a certain context should not necessarily be replicated elsewhere. Modifications of programs and sensitivity to regional cultural norms are necessary in future person-centered family planning intervention design and in the evaluation of program outcomes. This is especially important to consider in light of the fact that most of these interventions were implemented in the US and Europe.

Our review highlights several important implications for practice and policy related to the implementation and potential impact of person-centered family planning programs. First, this review provides descriptive evidence that person-centered family planning interventions have the potential to improve quality of care. Additionally, our review indicates that improvement in family planning knowledge in particular may be achievable with the implementation of person-centered family planning programs. Programs wishing to improve quality of family planning services can use our findings to guide program development and implementation so that the most effective programs in the context are implemented. The domains outlined and highlighted from the interventions may be used by programs who seek to improve specific aspects of quality of care in their facilities (i.e. supportive care, communication, autonomy, etc.).

In general, a number of gaps in the existing literature and directions for future person-centered family planning interventions have also been identified by our review. Specifically, there is a need for rigorous evaluations of the interventions measuring aggregate level outcomes and the long-term effects of person-centered programs on family planning uptake and use as well as fertility related outcomes. Our evidence generally supports positive outcomes on knowledge as well as quality of person-centered family planning programs, however more evidence is needed to understand the nuances of how and when family planning knowledge and quality of programming can lead to behavior change in the short and long term.

## References

[1] Ekman I, Swedberg K, Taft C, Lindseth A, Norberg A, Brink E (2011). Person-centered care — Ready for prime time. Eur J Cardiovasc Nurs.

[2] Millenson M, Berenson R. The road to making patient-centered care real: policy vehicles and potholes. Urban Institute; 2015. http://www.urban.org/sites/default/files/alfresco/publication-pdfs/2015.10.12_Millenson_Berenson.pdf. Accessed 3 Jun 2016.

[3] Nalwadda G, Mirembe F, Tumwesigye NM, Byamugisha J, Faxelid E (2011). Constraints and prospects for contraceptive service provision to young people in Uganda: providers’ perspectives. BMC Health Serv Res.

[4] Schuler SR, Hossain Z (1998). Family planning clinics through women’s eyes and voices: a case study from rural Bangladesh. Int Fam Plan Perspect.

[5] Tumlinson K, Speizer IS, Archer LH, Behets F (2013). Simulated clients reveal factors that may limit contraceptive use in Kisumu, Kenya. Glob Health Sci Pract.

[6] RamaRao S, Lacuesta M, Costello M, Pangolibay B, Jones H (2003). The link between quality of care and contraceptive use. Int Fam Plan Perspect.

[7] Dehlendorf C, Henderson JT, Vittinghoff E, Grumbach K, Levy K, Schmittdiel J (2016). Association of the quality of interpersonal care during family planning counseling with contraceptive use. Am J Obstet Gynecol.

[8] Abdel-Tawab N, Roter D (2002). The relevance of client-centered communication to family planning settings in developing countries: lessons from the Egyptian experience. Soc Sci Med 1982.

[9] Tunçalp Ӧ, Were W, MacLennan C, Oladapo O, Gülmezoglu A, Bahl R (2015). Quality of care for pregnant women and newborns—the WHO vision. BJOG Int J Obstet Gynaecol.

[10] Bohren MA, Vogel JP, Hunter EC, Lutsiv O, Makh SK, Souza JP (2015). The mistreatment of women during childbirth in health facilities globally: a mixed-methods systematic review. PLoS Med.

[11] Kruk ME, Kujawski S, Mbaruku G, Ramsey K, Moyo W, Freedman LP. Disrespectful and abusive treatment during facility delivery in Tanzania: a facility and community survey. Health Policy Plan. 2018;33(1):e26–e33. 10.1093/heapol/czu079. 29304252

[12] Abuya T, Warren CE, Miller N, Njuki R, Ndwiga C, Maranga A (2015). Exploring the prevalence of disrespect and abuse during childbirth in Kenya. PLoS One.

[13] Sudhinaraset M, Afulani P, Diamond-Smith N, Bhattacharyya S, Donnay F, Montagu D. Advancing a conceptual model to improve maternal health quality: The Person-Centered Care Framework for Reproductive Health Equity. Gates Open Research. 2017;1:1. 10.12688/gatesopenres.12756.1. 10.12688/gatesopenres.12756.1 PMC5764229 29355215

[14] World Health Organization (2014). The prevention and elimination of disrespect and abuse during facility-based childbirth.

[15] Campbell Collaboration. Campbell collaboration systematic reviews: policies and guidelines. Campbell Syst Rev. 2014;2014 https://campbellcollaboration.org/library/campbell-collaboration-systematic-reviews-policies-and-guidelines.html. Accessed 6 June 2016

[16] Warnock R, Sudhinaraset M, Diamond-Smith N, Montagu D, Treleaven E. Protocol: A systematic review of interventions to improve quality of delivery, family planning and abortion services. http://www.crd.york.ac.uk/PROSPEROFILES/25425_PROTOCOL_20150801.pdf. Accessed 6 Jun 2016.

[17] Hombrados JG, Waddington H (2012). Internal validity in social experiments and quasi-experiments: an assessment tool for reviewers.

[18] Brody C, Hoop T de, Vojtkova M, Warnock R, Dunbar M, Murthy P, et al. The effects of economic self-help group programs on Women’s empowerment: a systematic review. Campbell Syst Rev 2015;11. https://campbellcollaboration.org/library/women-empowerment-economic-self-help-programmes.html. Accessed 11 Nov 2015.

[19] Higgins JP, Green S, others. Cochrane handbook for systematic reviews of interventions. Wiley Online Library; 2008. https://training.cochrane.org/handbook. Accessed 25 July 2016.

[20] Mwaikambo L, Speizer IS, Schurmann A, Morgan G, Fikree F (2011). What works in family planning interventions: a systematic review of the evidence. Stud Fam Plan.

[21] Costello M, Lacuesta M, RamaRao S, Jain A (2001). A client-centered approach to family planning: the Davao project. Stud Fam Plan.

[22] Jain AK, Ramarao S, Kim J, Costello M (2012). Evaluation of an intervention to improve quality of care in family planning programme in the Philippines. J Biosoc Sci.

[23] Amatya R, Akhter H, McMahan J, Williamson N, Gates D, Ahmed Y (1994). The effect of husband counseling on NORPLANT contraceptive acceptability in Bangladesh. Contraception.

[24] Exner TM, Mantell JE, Adeokun LA, Udoh IA, Ladipo OA, Delano GE (2009). Mobilizing men as partners: the results of an intervention to increase dual protection among Nigerian men. Health Educ Res.

[25] Kraft JM, Harvey SM, Thorburn S, Henderson JT, Posner SF, Galavotti C (2007). Intervening with couples: assessing contraceptive outcomes in a randomized pregnancy and HIV/STD risk reduction intervention trial. Womens Health Issues.

[26] Sarnquist CC, Moyo P, Stranix-Chibanda L, Chipato T, Kang JL, Maldonado YA (2014). Integrating family planning and prevention of mother to child HIV transmission in Zimbabwe. Contraception.

[27] Gilliam ML, Martins SL, Bartlett E, Mistretta SQ, Holl JL (2014). Development and testing of an iOS waiting room “app” for contraceptive counseling in a title X family planning clinic. Am J Obstet Gynecol.

[28] Charron-Prochownik D, Sereika SM, Becker D, White NH, Schmitt P, Powell AB (2013). Long-term effects of the booster-enhanced READY-girls preconception counseling program on intentions and behaviors for family planning in teens with diabetes. Diabetes Care.

[29] Winter L, Breckenmaker LC (1991). Tailoring family planning services to the special needs of adolescents. Fam Plan Perspect.

[30] Schwandt HM, Creanga AA, Danso KA, Adanu RMK, Agbenyega T, Hindin MJ (2013). Group versus individual family planning counseling in Ghana: a randomized, noninferiority trial. Contraception.

[31] Berenson AB, Rahman M (2012). A randomized controlled study of two educational interventions on adherence with oral contraceptives and condoms. Contraception.

[32] Carneiro Gomes Ferreira AL (2011). Impieri Souza a, Evangelista Pessoa R, Braga C. The effectiveness of contraceptive counseling for women in the postabortion period: an intervention study. Contraception.

[33] Kim YM, Kols A, Putjuk F, Heerey M, Rinehart W, Elwyn G (2003). Participation by clients and nurse midwives in family planning decision making in Indonesia. Patient Educ Couns.

[34] Danielson R, Marcy S, Plunkett A, Wiest W, Greenlick MR (1990). Reproductive health counseling for young men: what does it do?. Fam Plan Perspect.

[35] León FR, Roca S, Ríos A, Zumarán A, Feijoo AR (2004). One-year client impacts of quality of care improvements achieved in Peru. Population Council, Frontiers in Reproductive Health.

[36] León FR, Brambila C, de la Cruz M, García Colindres J, Morales C, Vásquez B (2005). Providers’ compliance with the balanced counseling strategy in Guatemala. Stud Fam Plan.

[37] Schwarz EB, Burch EJ, Parisi SM, Tebb KP, Grossman D, Mehrotra A (2013). Computer-assisted provision of hormonal contraception in acute care settings. Contraception.

[38] Nobili MP, Piergrossi S, Brusati V, Moja EA (2007). The effect of patient-centered contraceptive counseling in women who undergo a voluntary termination of pregnancy. Patient Educ Couns.

[39] Petersen R, Albright J, Garrett JM, Curtis KM (2007). Pregnancy and STD prevention counseling using an adaptation of motivational interviewing: a randomized controlled trial. Perspect Sex Reprod Health.

[40] Sathar Z, Jain A, RamaRao S, ul Haque M, Kim J (2005). Introducing Client-Centered Reproductive Health Services in a Pakistani Setting. Stud Fam Plann.

[41] Kim YM, Putjuk F, Basuki E, Kols A (2000). Self-assessment and peer review: improving Indonesian service providers’ communication with clients. Int Fam Plan Perspect.

[42] Bensussen-Walls W, Saewyc EM (2001). Teen-focused care versus adult-focused Care for the High-Risk Pregnant Adolescent: an outcomes evaluation. Public Health Nurs.

[43] Reynolds HW, Toroitich-Ruto C, Nasution M, Beaston-Blaakman A, Janowitz B (2008). Effectiveness of training supervisors to improve reproductive health quality of care: a cluster-randomized trial in Kenya. Health Policy Plan.

[44] Langston AM, Rosario L, Westhoff CL (2010). Structured contraceptive counseling—a randomized controlled trial. Patient Educ Couns.

[45] Rawlins BJ, Kim Y-M, Rozario AM, Bazant E, Rashidi T, Bandazi SN (2013). Reproductive health services in Malawi: An evaluation of a quality improvement intervention. Midwifery.

